# Co-Delivery of Doxycycline and Hydroxychloroquine Using CdTe-Labeled Solid Lipid Nanoparticles for Treatment of Acute and Chronic Brucellosis

**DOI:** 10.3389/fchem.2022.890252

**Published:** 2022-05-11

**Authors:** Seyed Mostafa Hosseini, Abbas Farmany, Mohammad Yousef Alikhani, Mohammad Taheri, Sara Soleimani Asl, Saeed Alamian, Mohammad Reza Arabestani

**Affiliations:** ^1^ Department of Microbiology, Faculty of Medicine, Hamadan University of Medical Sciences, Hamadan, Iran; ^2^ Dental Research Center, School of Dentistry, Hamadan University of Medical Sciences, Hamadan, Iran; ^3^ Department of Anatomical Sciences, Faculty of Medicine, Hamadan University of Medical Sciences, Hamadan, Iran; ^4^ Razi Vaccine and Serum Research Institute, Agricultural Research, Education and Extension Organization (AREEO), Karaj, Iran; ^5^ Brucellosis Research Center, School of Medicine, Hamadan University of Medical Sciences, Hamadan, Iran

**Keywords:** brucellosis, *Brucella melitensis*, solid lipid nanoparticles, doxycycline, hydroxychloroquine

## Abstract

Brucellosis is a systemic disease in both acute and chronic forms which can affect any organ or tissue in the body. One of the biggest issues in treating this disease is its relapse. In this study, a complete treatment of brucellosis was evaluated using enhanced performance of doxycycline and hydroxychloroquine drugs by using solid lipid nanoparticles (SLN) conjugated cadmium-telluride quantum dots. The double emulsion method was used to prepare SLN and cadmium-telluride quantum dots. The physicochemical properties of NPs were determined. The effect of nanoparticle-loaded antibiotics against *Brucella melitensis* was determined by well diffusion, minimum inhibitory concentration (MIC), cell culture, and animal studies. The means of particle size, PDI, zeta potential, drugs loading, and encapsulation efficiency were 214 ± 25 nm, 0.385 ± 0.022, −18.7 ± 2.3 mV, 17.7 ± 1.5%, and 94.15 ± 2.6%, respectively. The results of FTIR and DSC showed that no chemical reaction occurred between the components of the NPs. The effect of free drug and NPs on bacteria was the same by well diffusion and MIC method. Drug-loaded NPs significantly reduced the number of CFUs in the cell line and acute and chronic brucellosis compared to the free drug. In conclusion, the synthesized nanoparticles were safe and green. With the slow release of the drug (100 h), the accumulation of the drug at the bacterial site increases and causes a greater effect on the *B. melitensis* and improves the disease of brucellosis. The use of synthesized nanodrugs in this study had promising therapeutic results.

## Introduction

Brucellosis is a bacterial disease caused by various species of *Brucella*, which mainly infects cattle, pigs, goats, sheep, and dogs (Kamga et al., 2020). Humans usually acquire the disease through direct contact with infected animals, eating or drinking of contaminated animal products, or inhaling airborne agents. Most cases are caused by eating unpasteurized milk or cheese from infected goats or sheep (Shoukat et al., 2017; Kamga et al., 2020). Brucellosis is one of the most widespread diseases between human and animal (zoonoses), which has serious consequences for public health in native areas. Lack of hygiene in animal husbandry and food transportation is one of the causes of brucellosis, which is still a risk to public health (Franc et al., 2018).

It is a systemic infection that can affect any organ or tissue in the body. The clinical signs are extremely nonspecific and show very variable manifestations. Brucellosis is caused by bacteria in the *Brucella* family, including *Brucella melitensis*, *Brucella abortus*, *Brucella suis*, and *Brucella canis*. Among these, *Brucella melitensis* and *Brucella abortus* are more important, causing brucellosis of varying severity in humans (Hull and Schumaker, 2018). The ability to cause disease in this genus of bacteria depends on their intracellularity. Upon entering the host cell, *Brucella* experiences a multi-stage intracellular cycle. These cycles occur with the help of bacterial effectors secreted by the type IV secretion systems (T4SSs). The bacterium enters the macrophage by endocytosis (Smith et al., 2020).

Antibiotics commonly used to treat brucellosis include aminoglycosides, tetracyclines, sulfonamides, and trimethoprim (Hayat et al., 2018). Low penetration, inactivation in the cell environment, or degradation by lysosomal enzymes cause ineffective antibiotics prescribed against intracellular bacteria (Yan et al., 2021). However, the wide distribution of these drugs does not reach the bacteria completely, and their presence in non-infectious tissues, apart from their inherent toxicity, may lead to adverse effects. There is also a relapse of the disease after treatment (Abd Elbaser and Mohammed, 2018). The intracellular concentration of antibiotics depends on the balance between the entry and exit of antibiotics, drug metabolism, and accumulation in various intracellular structures such as phagolysosomes and endosomes with different acidic pHs that reduce the activity of aminoglycosides (Majzoobi et al., 2018).

According to previous studies, acidic conditions within macrophages are necessary for the proliferation and survival of *Brucella* bacteria and play an important role in the virulence of the disease (Akova et al., 1999; Majzoobi et al., 2018). Therefore, changing the acidic pH of phagolysosomes and shifting it to alkaline pH can increase the effect of antibiotics on intracellular bacteria, especially *Brucella* (Akova et al., 1999). For this reason, the use of compounds such as hydroxychloroquine can change the acidic conditions to alkaline conditions within the cell, which both affects the virulence of the bacterium and provides the conditions for the effectiveness of prescription antibiotics such as streptomycin and doxycycline (Hashemi et al., 2012). It has been shown that the development of a new drug alone is not effective in treatment; the low solubility of some drugs in water and the low viability of new drug molecules is an issue (Boyd et al., 2019). Therefore, it is important to develop drug delivery systems that can overcome these issues (Arshad et al., 2021b; Rahdar et al., 2021). This carrier system must be non-toxic and have the capacity to accept a sufficient amount of the drug, in addition to being able to target and control the release of the drug (Imbuluzqueta et al., 2010; Arshad et al., 2021a). Solid lipid nanoparticles have been developed as a carrier system for many applications, and many drugs with various applications have been successfully incorporated into community lipid nanoparticles (Paliwal et al., 2020; Hasanein et al., 2021). This drug delivery system causes controlled release of the drug and increases the chemical stability of the trapped drugs.

Fluorescent materials can be used to ensure that synthesized nanocarriers enter the macrophages. At this time, cadmium-telluride quantum dot is widely used to label nanoparticles (Bardajee et al., 2018; Kadim, 2019). The aim of this study was to develop a new treatment method to improve the efficacy of antibiotics and minimize the use of conventional antibiotics in the brucellosis treatment.

## Methods

### Materials

Palm oil (Softisan 154 or S154), a gift from Condea (Witten, Germany), and stearic acid (Merck, Germany) were the lipids used in this study. Surfactants include the following: Poloxamer407 (Sigma Aldrich, St. Louis, MI, United States) and polioxiethylene-20-sorbitan monooleate (tween 80- Sigma Aldrich, St. Louis, MI, United States), lipo-soluble surfactants including Span 80 (Sigma Aldrich, St. Louis, MI, United States) and lecithin (Sigma Aldrich, St. Louis, MI, United States), doxycycline (Sigma Aldrich, St. Louis, MI, United States), and hydroxychloroquine sulfate (Sigma Aldrich, St. Louis, MI, United States). Sodium borohydride (NaBH4) (Merck, Germany), tellurium (Sigma Aldrich), cadmium salt (Scharlau, Spain), thioglycolic acid (TGA) (Merck, Germany), and distilled water were used throughout the study.

### Preparation of Cadmium-Telluride Quantum Dot (CdTe)

500 mg of NaBH_4_ powder is added to a 50-ml balloon on a magnetic stirrer with 7 ml of distilled water. Then during the flow of argon gas, 90 mg of tellurium powder was added to the solution. Initially, because the mixture inside the balloon reacts strongly with oxygen, the color of the solution turns purple, but with the flow of argon gas, the purple color disappears and the solution becomes colorless. 2 h later, the white sodium tetraborate powder precipitates. Next, cadmium salt solution in a 250-ml flask to which thioglycolic acid (TGA) was added was prepared. NaHTe solution is added drop-wise to the cadmium salt. The nanoparticles begin to form and grow in the balloon when the Te^2−^ and Cd^2+^ ions combine. To grow the clusters, the balloon is placed in a water bath at 90°C (Nguyen et al., 2019).

### Preparation of Solid Lipid Nanoparticles

The nanoparticle was synthesized by using the double emulsion/melt dispersion technique. Different percentages of lipids, surfactants, and drugs were tested to achieve the optimal formulation. First emulsions included palm oil (1,200 Mg) or stearic acid, poloxamer407 (120 Mg), soy lecithin, distilled water, and antibiotics. Briefly, palm oil or stearic acid were heated at 70°C (5°C above the melting point) and 75°C (5°C above the melting point) using a ben murray, respectively. At this time, poloxamer407, soy lecithin, doxycycline (50 Mg) and hydroxychloroquine (75 Mg) antibiotics were added to the melted oil. It was mixed for 5 min under magnetic stirring (60°C and 150 rpm). Then, 1 ml heated distilled water was added to the mixture and homogenized. To obtain the first emulsion, the mixture was sonicated by using a sonicator (skymen, China) at 45% amplitude (20 W) for 60 s (W1/O). In the following step, 20 ml heated tween-80 was added to the first emulsion and the mixture was homogenized using an ultrasonic device (Bandelin Sonopuls, Berlin, Germany) at 45% amplitude (20 w) at a regular pulse rhythm (10 s on and 5 s off) for 60 s to the second emulsion (W1/O/W2) be provided. The second emulsion was added drop-wise to cold distilled water (4°C) containing 100 µl cadmium-telluride quantum dot (5 nm) under the magnetic stirring condition (5 min) to stabilize the synthesized nanoparticles. The exact same conditions were done for preparation of free SLN (solid lipid nanoparticles without drug). Materials and conditions for each formulation are presented in Table 1. Finally, the synthesized nanoparticles was separated using a high-speed centrifuge (35,000 rpm for 15 min) and washed three times using distilled water.

500 mg of each formulation was added to 1 ml glycerin and lyophilized using a vacuum pump (Christ, China) with a condenser flow at −80°C. To use lyophilized nanodrugs in biological studies on bacteria, the nanoparticles were first dissolved in distilled water and then sterilized by using a 450-nm filter (Black Peres et al., 2016; Hosseini et al., 2019a).

### Nanoparticle Characterization

The mean particle size, polydispersity index (PDI), and zeta potential of solid lipid nanoparticles loaded with doxycycline and hydroxychloroquine (DOX-HCQ-SLN) were measured at 25°C by using the dynamic light scattering (DLS) technique performed using a zetasizer nano ZS 3600 (Malvern Instruments, Worcestershire, United Kingdom) device. Each reported value was an average of three measurements. The mentioned factors were measured in two stages, first, immediately after preparation, and in the second stage, after lyophilization.

### Determine the Lambda Max (λ_Max_) and Standard Curve of Drugs

It is necessary to obtain the maximum adsorption wavelength of purchased drugs, in order to evaluate the efficiency of encapsulation and loading of drugs. For this purpose, 5 mg of each drug was weighed and dissolved in 5 ml of their solvent (distilled water for doxycycline and ethanol for hydroxychloroquine). The volume of 1 ml of this solution was increased to 10 ml with double distilled water (stock prepared: 100 μg/ml), and then serial dilutions of the stock solution were prepared using distilled water and antibiotic solvent. The absorbance (OD) of the samples was measured using a UV–vis spectrophotometer (UV-2100, Spectrum Technologies, Fort Worth, TX, United States) at wavelengths of 200–400 nm at 5 nm intervals and the maximum wavelength was recorded. Dilutions of 0–45 μg/ml (at 5 μg/ml intervals) were prepared in double distilled water using the prepared stock (10 mg of drug in 2 ml of solvent). Then the absorbance of each sample at maximum wavelength was read using a UV–vis spectrophotometer (Iorhemen et al., 2017). Then, the standard curve of each drug was drawn based on the obtained data.

### Encapsulation Efficacy and Drug Loading

To evaluate how much drug is loaded and encapsulated in the synthetic nanoparticle, direct and indirect methods were used. In the indirect method, 10 mg of the synthesized nanoparticle was dissolved in 10 ml of distilled water and completely vortexed to become homogeneous. 2 ml of the prepared suspension was centrifuged at 15,000 rpm for 30 min at 4°C, and this was done three times to make the supernatant completely clear. After centrifugation, the supernatant absorbance (OD) was read using a UV–vis spectrophotometer at wavelengths of 270 and 343 nm for doxycycline and hydroxychloroquine, respectively. The absorbance (OD) obtained was entered into the standard curve (previously plotted for drugs), and the amount of encapsulation and loading was calculated using the following formula (Gupta et al., 2016).

In the direct method, the amount of doxycycline and hydroxychloroquine contained in NPs was investigated by HPLC equipped (SY-8100, China) with a UV detector at a wavelength of 270 and 343 nm. The mobile phase was a mixture of water (50%) and ethanol (50%), the pump was of SY-8100 type, the injector loop was at 772 PSI, and the column was of C-18 type (4.6 × 250 mm), run at a flow rate of 1 ml/min. In this step, 10 mg of the sample alongside 10 ml of water and ethanol mixture were mixed and vortexed using sonication for 10 min to become homogenous. Then, it was centrifuged at 1,500 rpm for 20 min and the supernatant was purified using 220-nm filters (Chen et al., 2019). A linear calibration curve with a good correlation coefficient (*r* ^2^ = 0.9990) for concentrations in a range from 1 μg/ml to 250 μg/ml was achieved.

$$Entrapment\;Efficiency\;EE\left(\%\right)=\frac{initial\;drug\;amount\;-\;free\;drug\;amount\;}{initial\;drug\;amount}\times 100,$$

$$Drug\;Loading\;DL\left(\%\right)=\frac{initial\;drug\;amount\;-\;free\;drug\;amount}{initial\;lipid\;amount}\times 100.$$

### DSC Analysis

The thermal behavior of the optimal formulation and its components were evaluated using the device (METTLER TOLEDO—DSC 1). 5–10 mg of lyophilized powder of optimal formulation and each of its components including doxycycline, hydroxychloroquine, palm oil, and physical mixture of drug and lipid was examined in the temperature range of 20–400°C (5 k/min) and under nitrogen gas (80 ml/min), separately (Müsellim et al., 2018).

### FTIR Analysis

In order to investigate the chemical structure of the samples, the synthesized nanoparticle lyophilized powder (optimal formulation), and the nanoparticle components were mixed separately with potassium bromide (KBr) simultaneously and converted into compact discs using a hydraulic compressor. Spectroscopy was performed by FTIR spectroscopy (Perkin Elmer, Waltham, MA, Spectrum 400) in middle-range IR (4,000–400 cm^−1^) (Müsellim et al., 2018).

### Evaluation of Drug Release

Release was performed using a dialysis bag (with fine pores of 2.5 nm and molecular weight of 12,000–14,000 Daltons). The membrane was soaked twice in distilled water 12 h before use. Phosphate buffer with pH: 7.4 at 37°C was used as the release medium. Ten milligram of lyophilized powder of the optimal formulation was dispersed in 2 ml of double distilled water and placed in the bag, and both ends of the bag were sealed. Similarly, 10 mg of both free drugs were placed in 2 ml of double distilled water in a separate bag. The bags were placed separately in the release medium (60 ml), and the lids of the containers containing the bag and buffer were closed to prevent water vapor from escaping. Each vessel was then placed in a shaker incubator (37°C, 100 rpm). At constant intervals (0, 1, 2, 4, 20, 40, 60, 80, 100, 120, and 140 h), 2 ml was removed from the medium and immediately replaced with the same volume of release medium to maintain the sink condition. Drug released from the synthesized nanodrug was measured using a UV–vis spectrometer at the maximum wavelength of each drug (Yu et al., 2019).

### Physical and Chemical Stability of NPs

The stability of the optimal formulation in suspension was evaluated for the short term (1 week in terms of appearance and 1 month in terms of appearance and physicochemical properties) and long term (6–12 months in terms of appearance and physicochemical properties). In the short term, at intervals of 24, 48, 72, 96, 120, and 144 h, the samples were inspected visually. At this stage, formulation without sediment formation, drug leakage, and accumulation were considered as stable in the short term. To evaluate the stability of the optimal formulation over a long period of time, physical appearance and physicochemical parameters (particle size, PDI, and zeta potential) with respect to zero time over a long period (2–12 months) were evaluated at refrigerator temperature (5°C) (Kalpana and Devi Rajeswari, 2018).

### Morphology

A field emission scanning electronic microscope (Fe-SEM) (MIRA3, TESCAN Company, Brno, and Czech Republic) was used to examine the morphology of the nanoparticles. 2 mg of lyophilized nanoparticles were added to 2 ml of distilled water and sonicated. Then 2 μl of the suspension was transferred to a microscope slide and allowed to dry, covered with a thin layer of gold, and evaluated (Brodusch et al., 2018).

### Bacterial Strain, Cell Line and Rat

For *in vitro* and *in vivo* tests, *Brucella melitensis* 16M (purchased from Razi Vaccine and Serum Research Institute, Iran) was used. *Brucella* agar, TSB (Tryptic Soy Broth), and TSA (Tryptic Soy Agar) were used for bacterial culture at 37°C and 5% of CO_2_. The J774-A1 mouse macrophage cell line (purchased from the Pasteur Institute of Iran, Biology Bank) was employed for the cell culture study. Dulbecco’s Modified Eagle cell culture Medium (DMEM) was used with 10% FBS. Male Wistar rats (6–8 weeks old) weighing 250 (±30) gr were purchased from Hamadan University of Medical Sciences.

### Minimum Inhibitory Concentration, Well Diffusion

The well diffusion method and minimum inhibitory concentration of nanoparticles and free drug were performed according to CLSI guidelines. Concentrations of 12.5, 25, and 50 μg/ml were prepared from drugs and nanodrugs. 0.5 McFarland suspension of *Brucella melitensis* were cultured on Mueller-Hinton agar plates using sterile swabs. In the following step, Wells 8 mm in size were created in culture media by using a sterile Pasteur pipette and each well was filled in by 100 μl of nanodrugs and free drug and incubated at 37°C for 24, 48, and 72 h. After these time intervals, bacterial growth around the wells was measured for each well and used as a basis for analyzing the antibacterial performance of nanodrugs (Hosseini et al., 2017).

96-well flat-bottomed plates were used to determine the minimum concentration of drug and nanodrug that had the ability to inhibit bacterial growth. The first concentration which was used was the lowest amount of antibiotics and nanodrugs obtained in the well diffusion test. 100 μl was added to each well from different dilutions. Then 100 μl of mueller hinton broth culture medium was added to each well, and finally, 5 μl of 0.5 McFarland bacterial suspensions was added to all wells and incubated for 24, 48, and 72 h at 37°C. After this time, to determine the minimum concentration, it was first examined visually, and in wells where bacteria had not grown, it was considered as the minimum inhibitory concentration (Espinel-Ingroff et al., 2017).

### MTT Assay

The MTT kit (3- (4, 5-Dimethylthiazol-2-yl) -2, 5-diphenyltetrazolium bromide) was used to evaluate the cytotoxicity of the synthesized nanodrug according to the instructions of the kit and Mouse monocyte-macrophage cells J774A.1 (ATCC TIB-67; BALB/c Mouse, hematopoietic, macrophage-like. 1 × 10^4^ cells were added to each well containing DMEM (Gibco Product, Carlsbad, CA) enriched by 10% of FBS and 1% of penicillin–streptomycin antibiotics and incubated for 24 h at 37°C and 5% of CO_2_. After this period, the medium was removed from the wells and 100 μL of Free doxycycline, Free hydroxychloroquine, DOX-SLN, HCQ-SLN, and Free SLN (blank sample) at various concentrations (25, 50, 100, 200, 400, and 800 μg/ml) alongside DMEM containing 10% FBS were added and incubated for 24 h.

The treated cells were washed with PBS, and 150 μl of fresh DMEM medium without fetal bovine serum was added to the wells again. 10 μl of MTT assay reagent was added and the culture medium was removed after 3–4 h of incubation, and then 200 μl of DMSO was added to each well and placed on a shaker at 100 RPM for 20 min to dissolve the formazan particles. Optical absorption of cell-containing plates was evaluated at 570 nm. The number of viable cells in each well was evaluated by positive control comparison (Sánchez-López et al., 2016; Kumar et al., 2018; Fernandes et al., 2021).

### *In Vitro* Cellular Infection

24-cell culture plates (Corning Inc., Corning, NY, United States) were used to investigate the effect of nanoparticles on *Brucella melitensis*. 10^5^ cells were added to each well containing DMEM medium with 10% of FBS and incubated for 24 h. Then, cells were infected with *Brucella melitensis* in log phase at a 1:100 ratio of cell to bacteria and incubated for 1 h to bacterial phagocytosis. The cells were washed three times with DMEM medium containing 50 μg/ml gentamicin antibiotic (Sigma Product Co.) to remove bacteria that were non-phagocyted or attached to the cell wall. Different dilutions of nanoparticles and drug (25, 50, and 100 μl) along with DMEM medium with 10% FBS were added to the cells and incubated for 24, 48, and 72 h at 37°C and 5% of CO_2_. After these times, the cells were washed using PBS twice. The cells were lysed with 250 μl of 0.1% Triton X-100 in order to determine the number of bacteria in the macrophages. 10 serial dilution was prepared from this suspension, and cultured on *Brucella* agar medium. The colony-forming unit (CFU) of bacteria was numbered and recorded after 24, 48, and 72 h of culture (Hosseini et al., 2019b; Hosseini et al., 2020).

### *In Vivo* Studies: Animal Experiment

Rats were infected with 1 ml of 1.5 × 10^6^ CFUs of *Brucella melitensis* M16 by intraperitoneal injection. Animal studies were performed in two phases of acute and chronic brucellosis. Rats were divided into eight groups after 10 days of bacterial injection (acute phase) and 5 weeks after injection (chronic phase). All experiments were done according to the guidelines for maintenance, surveillance, and usage of laboratory animals published by the National Institute of Health United State (NIH publication No. 85-23, revised 1985). Moreover, the study was approved by the ethics committee of the Hamadan University of Medical Sciences (No: IRUMSHA. REC.1399.736). Three and five rats were considered in each group, for acute and chronic phases of the disease, respectively. The dose of injection was based on rat weight. Study groups included the following: 1) control group (infected without treatment), 2) DOX-SLN-treated group (2.5 mg/kg), 3) HCQ-SLN-treated group (6.5 mg/kg), 4) DOX-HCQ-SLN–treated group (4.5 mg/kg), 5) free hydroxychloroquine–treated group (6.5 mg/kg), 6) free doxycycline–treated group (2.5 mg/kg), 7) free doxycycline and free hydroxychloroquine–treated group (4.5 mg/kg), and 8) drug-free nanoparticle-treated group. In the acute phase of brucellosis, three doses of drug and nanoparticle were administered every other day (days 11, 13, and 15 after bacterial injections). In the chronic phase of brucellosis, ten doses of drugs and nanoparticle were administered intraperitoneally 5 weeks after bacterial injection (once daily).

One day after the last injection of the drug and nanodrugs, the rats were euthanized. The liver and spleen were extracted and weighted in observance of sterile conditions. Tissues were homogenized using sterile physiological serum. Different dilutions were prepared from the suspension and cultured on *Brucella* agar medium and incubated for 4 days at 37°C with 5% of CO_2_. The number of bacterial colonies that had grown was then counted and recorded (Hosseini et al., 2019b; Ghaderkhani et al., 2019).

### Statistical Analysis

Due to the fact that our data are non-parametric, Kruskal–Wallis test was used to compare the number of bacterial colonies grown between groups in the acute and chronic stages. All statistical tests were performed at the 0.05 level of confidence.

## Results

### Physicochemical Characterization of Nanoparticles (Size, PDI, and Zeta Potential)

**FIGURE 1:**
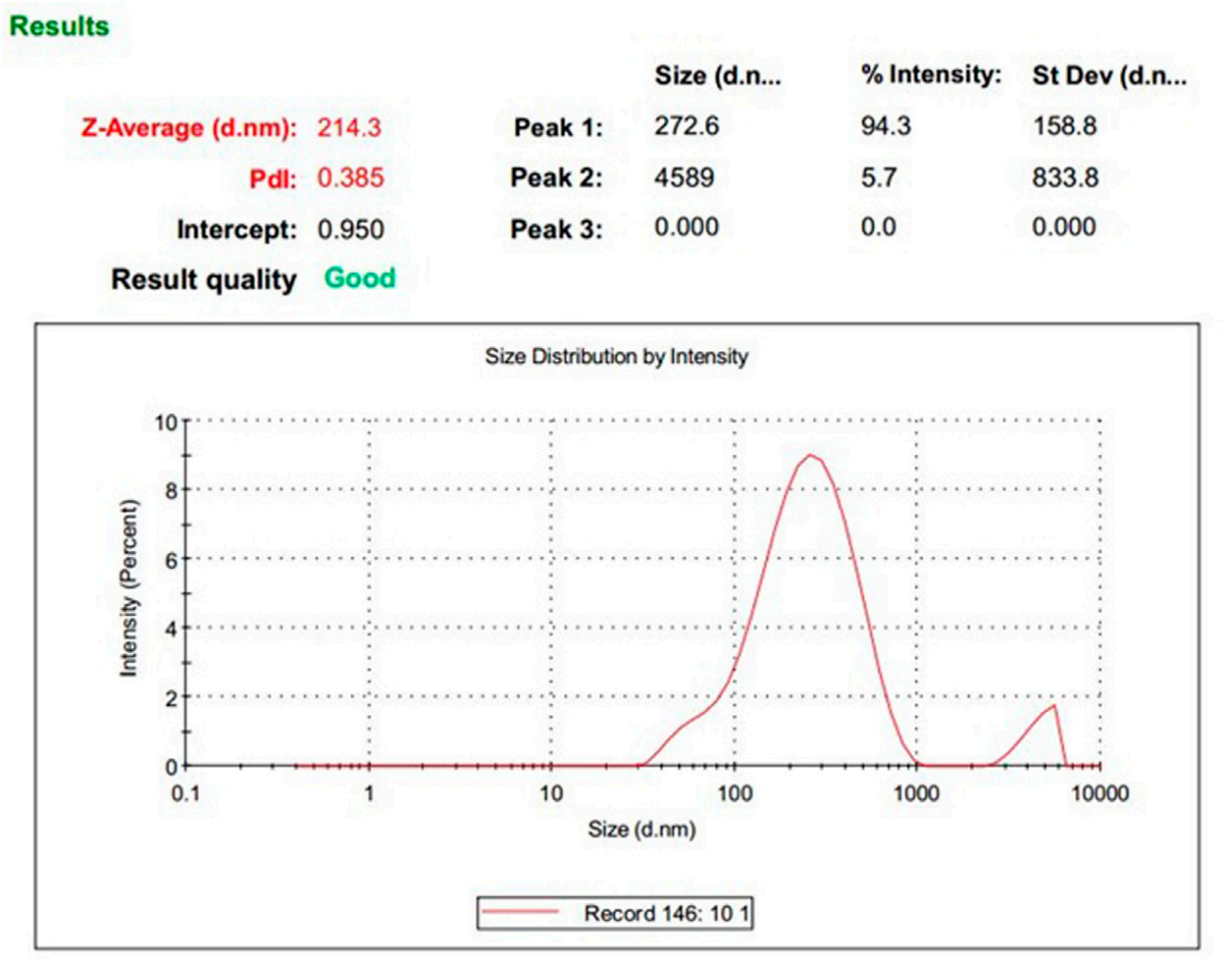
DLS results: size and PDI of DOX-HCQ-SLN.

**FIGURE 2:**
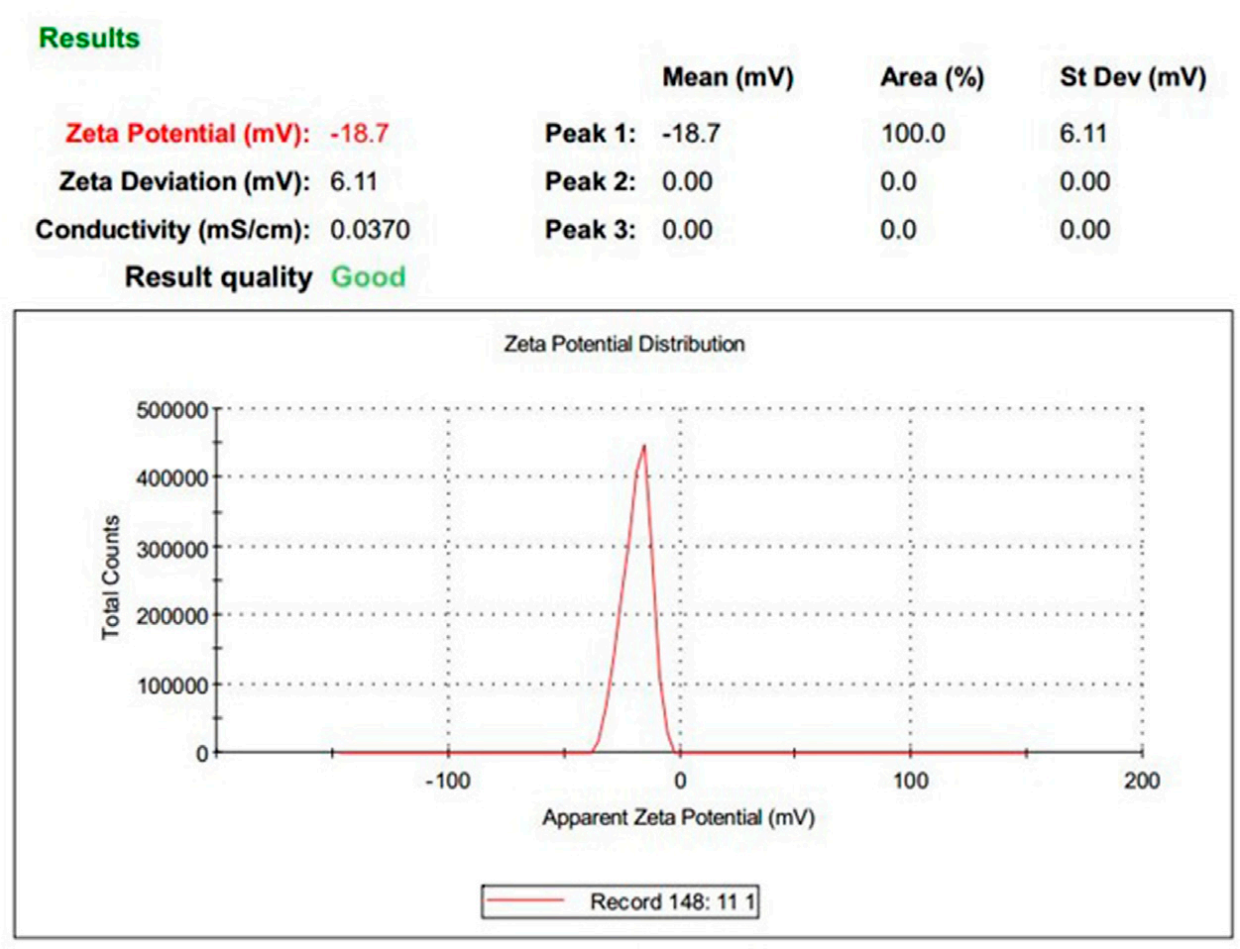
DLS results: zeta Potential of DOX-HCQ-SLN.

Depending on the lipid and surfactant used, the duration of homogenization, and strength of the ultrasound probe, the properties of nanoparticles differ (Table 1). The mean size and optimum PDI for the nanoparticles used for the rest of the study were 214.3 ± 25 and 0.385 ± 0.02, respectively. The zeta potential of the optimum nanoparticle was −18.7 mV (Figures 1, 2).

**TABLE 1:** Materials used in some formulations.

Formulation	Doxycycline (mg)	Hydroxychloroquine (mg)	Palm oil (mg)	Stearic acid (mg)	Poloxamer 407 (mg)	Lecithin (mg)	Tween 80 (ml)	Water added (ml)
F1	10	15	200	—	60	—	10	20
F2	20	25	—	200	—	60	10	20
F3	30	40	600	—	80	—	20	30
F4	40	50	—	600	—	80	20	30
F5	**50**	**75**	**1,200**	—	**120**	—	**30**	**50**
F6	60	90	—	1,200	—	120	30	50
F7	75	110	1,500	—	150		40	75
F8	75	110	—	1,500	—	150	40	75

They mean that optimum condition for formulation.

### Encapsulation and Drug Loading

The encapsulation rate of doxycycline and hydroxychloroquine in the synthesized nanoparticles was 95.8 and 92.5%, respectively. The mean drug load in different formulations was 12.79%. In optimum formulation, the amount loaded was 17.7% (Table 2).

**TABLE 2:** Properties of DOX-HCQ-SLN

	Formulation	PS (nm)	PDI	Zeta potential (mV)	Doxycycline encapsulation (%)	Hydroxychloroquine encapsulation (%)	Doxycycline loading (%)	Hydroxychloroquine loading (%)
Before lyophilization	F1	114.3	0.246	−21.5	95.5	90.1	15.4	15.1
	F2	155.8	0.294	−15.6	98.8	95.0	14.1	13.2
	F3	280.4	0.452	−19.0	96.2	88.3	13.2	12.9
	F4	**221.1**	**0.333**	**−22.2**	**95.3**	**85.6**	**16.9**	**15.5**
	F5	210.3	0.343	−19.5	97.3	91.6	14.8	11.1
	F6	200.8	0.299	−17.7	92.4	85.8	12.2	10.0
	F7	293.4	0.390	−15.9	91.3	89.9	14.6	9.9
	F8	430.8	0.456	−19.9	95.9	91.6	11.3	9.8
After lyophilization	F1	120.4	0.270	22.2	93.2	90.2	15.3	14.3
	F2	170.9	0.365	16.6	95.5	88.3	10.2	9.6
	F3	300.5	0.456	20.5	90.2	84.7	14.9	11.3
	F4	**246.8**	**0.398**	**25.6**	**95.8**	**92.5**	**19.1**	**16.3**
	F5	214.3	0.385	−18.7	96.1	90.0	14.5	9.5
	F6	232.1	0.342	−13.9	93.4	87.9	12.1	10.9
	F7	302.5	0.401	−16.9	90.2	88.3	13.2	11.8
	F8	436.8	0.55	−21.5	91.1	87.6	11.9	9.8

They mean that optimum condition for formulation.

### FE-SEM Microscopy

**FIGURE 3:**
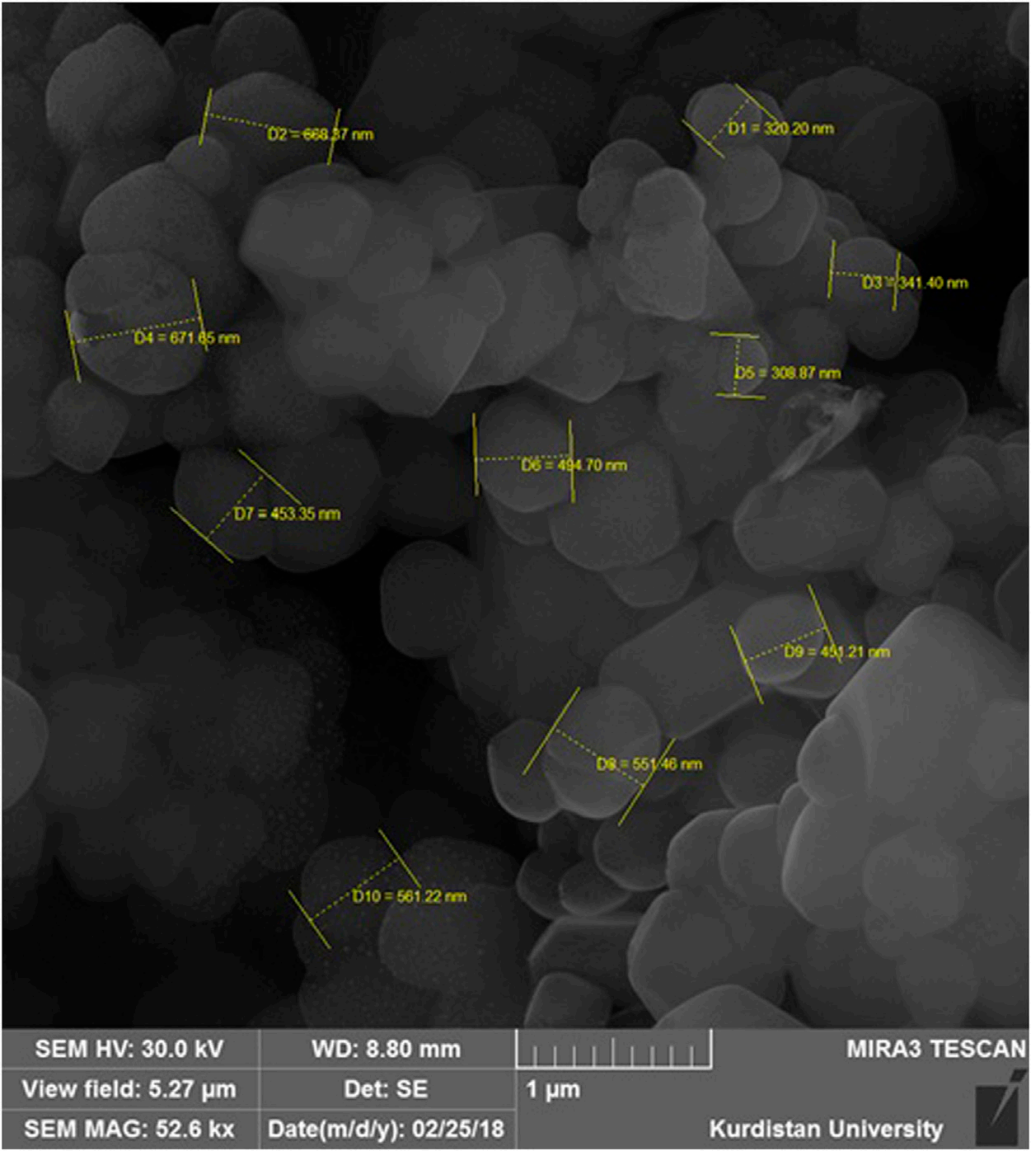
Field emission scanning electronic microscope images of DOX-HCQ-SLN.

The results of electron microscopy showed that the synthesized nanoparticles were morphologically spherical and had a smooth surface with homogeneous polydispersity (Figure 3).

### DSC Analysis and Fourier-Transform Infrared Spectroscopy Analysis

**FIGURE 4:**
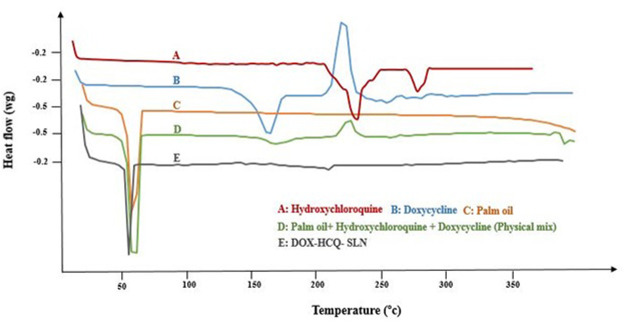
DSC thermograms of nanoparticle components.

DSC analysis was used to investigate the melting behavior of DOX-HCQ-SLN. Thermograms of DOX-HCQ-SLN, palm oil, free doxycycline, free hydroxychloroquine, and [free doxycycline and free hydroxychloroquine (physical mixture)], shown in Figure 4. As shown in Figure 4, the DSC thermogram shows the melting process of palm oil at 63°C. The melting points of the physical mixture and DOX-HCQ-SLN contained cadmium-telluride quantum dots are similar to the melting point of the palm oil. In the DSC thermograms of doxycycline and hydroxychloroquine, a sharp endothermic peak was seen at 230 and 250°C. While these points in other compounds show a small melting point for physical mixture and DOX-HCQ-SLN. Note that no any significant change was observed in the endothermic peak positions of doxycycline, hydroxychloroquine, physical mixture, and DOX-HCQ-SLN. The absence of a sharp melting peak of DOX-HCQ-SLN thermogram suggests that no free drug crystal remained in the synthesized nanoparticles.

**FIGURE 5:**
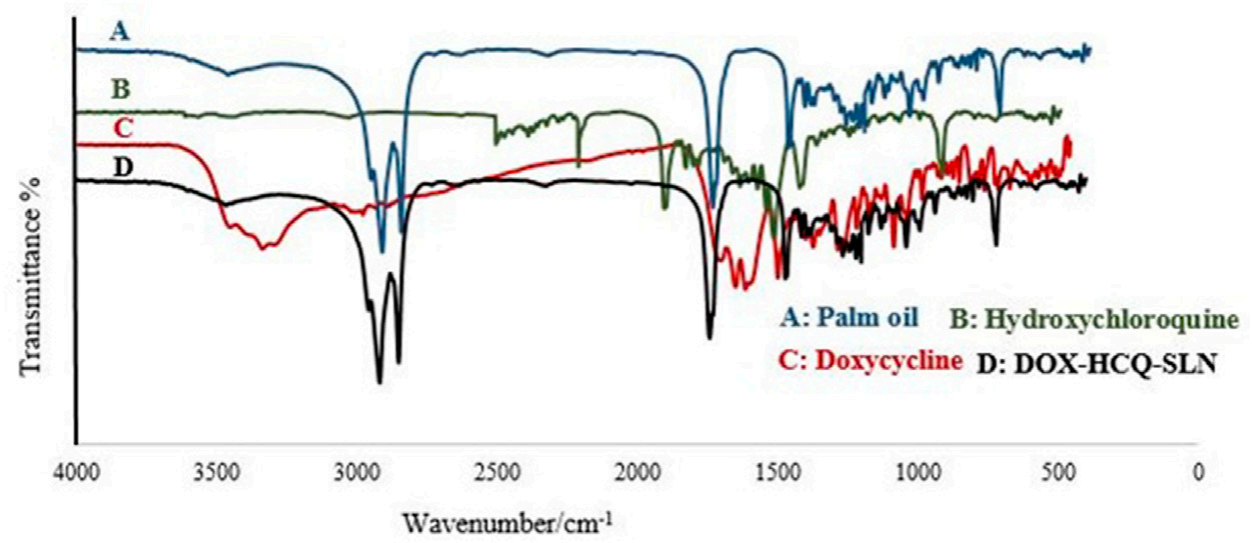
FTIR spectra of nanoparticle components.

The FTIR spectra of doxycycline, hydroxychloroquine, palm oil, and DOX-HCQ-SLN are shown in Figure 5. According to Figure 5, it can be seen that DOX-HCQ-SLN absorption peaks are consistent with the basic ingredients of nanoparticles (palm oil, antibiotics, and surfactant), and new absorption peaks were not seen indicating a new functional group or bond. However, a small change in the oil melting process was observed in the physical mixture.

### Nanoparticle Stability

Examination of stability of nanoparticles at different times showed that the synthesized optimum formulation was still stable after 6 months. After 9 months, the synthesized nanoparticles did not change in terms of color and turbidity. However, an increase in PDI was observed. The size also changed from 214.3 to 258.3 (20.53%) Table 3.

**TABLE 3:** Stability of DOX-HCQ-SLN

	Time (months), 4°C				
	0	1	3	6	9
Average diameter nm (±SD)	214.3 ± 27	213.6 ± 25	225.6±	230.5 ± 31	258.3 ± 34
Polydispersity index	0.385	0.390	0.410	0.405	0.412
Zeta potential (mV ± SD)	−18.7 ± 2.5	−17.5 ± 2.3	−19.9 ± 2.7	−17.6 ± 2.6	−18.3 ± 2.5
Appearance (color or turbidity)	Light yellow, no turbidity	Light yellow, no turbidity	Light yellow, no turbidity	Light yellow, no turbidity	Light yellow, no turbidity

### Drug Release

**FIGURE 6:**
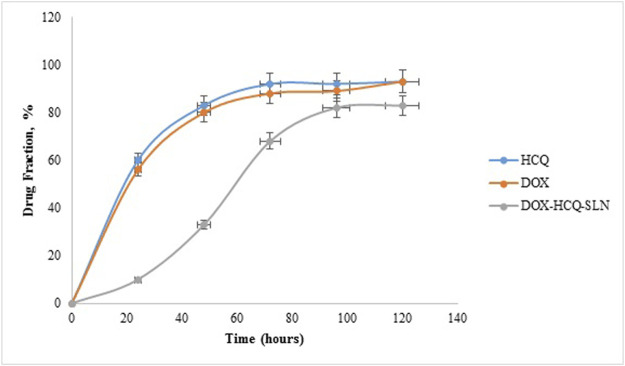
Release test of DOX-HCQ-SLN, free doxycycline, and hydroxychloroquine.

The release test was performed for Free DOX, Free HCQ, and DOX-HCQ-SLN over a period of 120 h (pH: 7.4, PBS buffer). In the first 20 h, there was a very rapid release in free DOX and free HCQ (about 60% of the total drug). During this time, only 10% of the drug was released from DOX-HCQ-SLN. Overall, it took 100 h for 82% of the drug to be released from DOX-HCQ-SLN (Figure 6).

### Toxicity of DOX-HCQ-SLN

**FIGURE 7:**
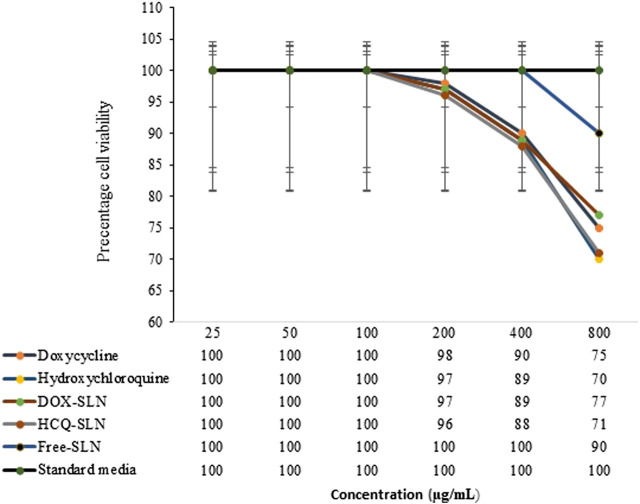
Effect of NPs and free drugs on J774A.1 cells.

The results of toxicity study showed that up to a concentration of 100 μg/ml of various formulations had no toxic effect on cells. In this study, there was no significant difference between synthesized nanoparticles with free drug in terms of effect on cells (*p* > 0.5). Free SLN, which included palm oil, poloxamer407, and Tween 80, had no toxic effect on cells up to 400 mg/ml (Figure 7). The dose used in antibacterial studies in *in vitro* and *in vivo* conditions was much lower than the concentrations used in the toxicity study.

### Antibacterial Study

**FIGURE 8:**
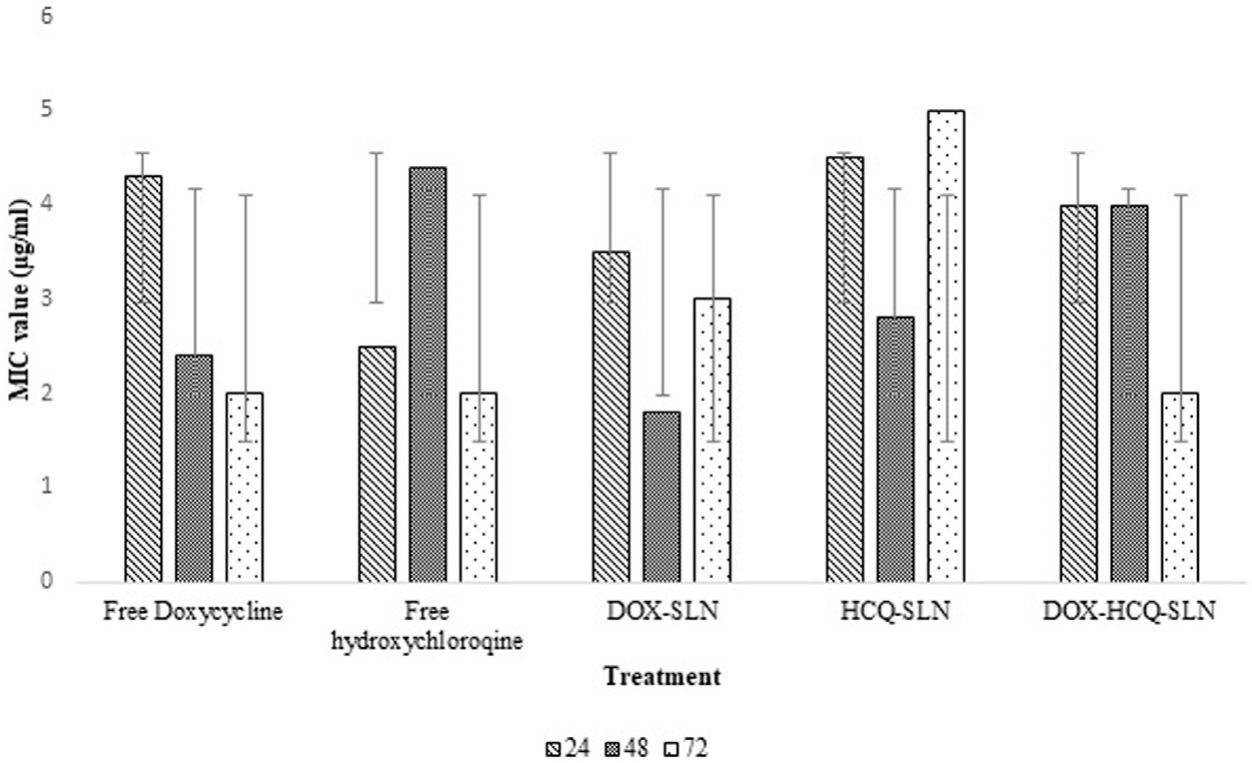
Results of MIC test in different times.

Different concentrations of free drugs and nanodrugs were used in this experiment. Well diffusion and MIC findings showed that there was no significant difference between nanodrugs and free drug in terms of inhibiting bacterial growth (*p* > 0.5). Free DOX and Free HCQ performed better than DOX-HCQ-SLN in the first 24 h. Bacterial inhibition in 72 h after treatment was approximately the same for DOX-HCQ-SLN and Free drugs. The minimum inhibitory concentrations of DOX-HCQ-SLN and free drugs were close after 72 h and there was no statistically significant difference (*p* > 0.05, Figure 8).

### Intracellular Study

**FIGURE 9:**
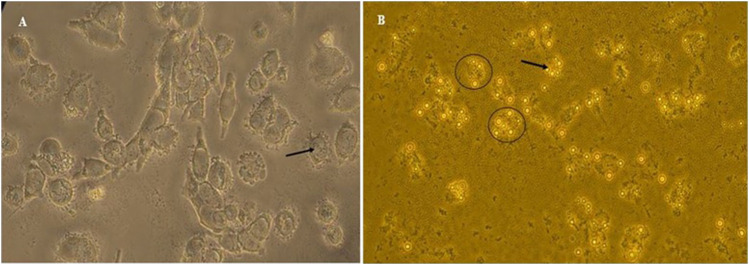
**(A)** Bacteria inside the macrophage. **(B)** Labeled NPs inside the macrophage.

The results of cell treatment with different concentrations of free drugs and nanodrugs showed that DOX-HCQ-SLN had the greatest effect on bacteria enclosed macrophages. The use of 100 μg/ml reduced the number of bacterial colonies to 3.5 log compared to the control group, which reduced it by 6.5 log. Comparison of treatment groups showed that there was a significant difference between DOX-HCQ-SLN and DOX-SLN in reducing the number of bacteria with Free DOX-HCQ and Free DOX (*p* < 0.5). Importantly, Free HCQ and HCQ-SLN alone had little effect on reducing the number of bacteria (Table 4). The entry of the bacteria and labeled nanoparticles into the cell is shown in Figure 9.

**TABLE 4:** Comparison between free drugs and NPs against *B. melitensis* inside J774A.1 cells.

Treatment	Concentration (µg/ml)									
	100		50		25		12.5		6.25	
	Mean CFUs ±SEM	Log CFUs reduction	Mean CFUs ±SEM	Log CFUs reduction	Mean CFUs ±SEM	Log CFUs reduction	Mean CFUs ±SEM	Log CFUs reduction	Mean CFUs ±SEM	Log CFUs reduction
Free SLN	6.5 ± 0.07	0	6.5 ± 0.07	0	6.5 ± 0.07	0	6.5 ± 0.07	0	6.5 ± 0.07	0
Free Doxycycline	5.5 ± 0.03	1	5.7 ± 0.02	0.8	5.8 ± 0.01	0.7	6.0 ± 0.03	0.5	6.1 ± 0.04	0.4
Free Hydroxychloroquine	6.4 ± 0.01	0.1	6.4 ± 0.03	0.1	6.4 ± 0.09	0.1	6.4 ± 0.01	0.1	6.4 ± 0.06	0.1
DOX-SLN	4.2 ± 0.04	2.3	4.5 ± 0.05	2.0	4.9 ± 0.01	1.6	5.1 ± 0.07	1.4	5.2 ± 0.04	1.3
HYD-SLN	6.4 ± 0.01	0.1	6.4 ± 0.05	0.1	6.4 ± 0.03	0.1	6.4 ± 0.06	0.1	6.4 ± 0.05	0.1
DOX-HYD-SLN	3.5 ± 0.07	3	3.7 ± 0.02	2.8	3.8 ± 0.05	2.7	4.1 ± 0.07	2.4	4.9 ± 0.09	1.6
Negative control	6.5 ± 0.05	0	6.5 ± 0.05	0	6.5 ± 0.05	0	6.5 ± 0.05	0	6.5 ± 0.05	0

## Results of Animal Studies

The number of bacterial colonies in the spleen and liver was counted in the acute and chronic stages of the disease. There was a statistically significant decrease in the number of bacterial colonies in the spleen and liver between the treated groups DOX-HCQ-SLN, DOX-SLN, Free Doxycycline, and Free DOX-HCQ (Mix) compared to the untreated group in the acute phase (*p* < 0.5). HCQ-SLN did not reduce the number of bacterial colonies compared to the untreated group (*p* > 0.05, Table 5). In the chronic phase, unlike the acute phase of the disease, DOX-HCQ-SLN had the greatest effect on reducing the number of bacterial colonies in the spleen and liver. This difference was statistically significant compared to other groups (*p* < 0.05, Table 6).

**TABLE 5:** *In vivo* efficacy of NPs and free drugs against *B. melitensis* in the acute phase.

Treatment	Acute stage			
	CFUs (Log_10_) in spleen	Log CFUs reduction	CFUs (Log_10_) in liver	Log CFUs reduction
Untreated (control)	4.1 ± 0.02	0.0	3.9 ± 0.03	0.0
DOX-SLN	3.1 ± 0.08	1.0	2.9 ± 0.01	1.0
HCQ-SLN	4.01 ± 0.06	0.09	3.8 ± 0.05	0.1
DOX-HCQ-SLN	2.5 ± 0.03	1.6	2.1 ± 0.01	1.8
Free doxycycline	3.7 ± 0.02	0.4	3.5 ± 0.04	0.4
Free hydroxychloroquine	4.0 ± 0.03	0.1	3.8 ± 0.01	0.1
Free DOX-HCQ (Mix)	3.5 ± 0.06	0.6	3.2 ± 0.05	0.7
Free SLN	4.02 ± 0.09	0.08	3.8 ± 0.05	0.1

**TABLE 6:** *In vivo* efficacy of NPs and free drugs against *B. melitensis* in the chronic phase.

Treatment	Chronic stage			
	CFUs (Log_10_) in spleen	Log CFUs reduction	CFUs (Log_10_) in liver	Log CFUs reduction
Untreated (control)	3.2 ± 0.03	0.0	2.8 ± 0.04	0.0
DOX-SLN	2.3 ± 0.04	0.9	1.9 ± 0.03	0.9
HCQ-SLN	3.02 ± 0.03	0.18	2.7 ± 0.02	0.1
DOX-HCQ-SLN	1.6 ± 0.01	1.6	1.3 ± 0.04	1.5
Free doxycycline	2.8 ± 0.05	0.4	2.4 ± 0.07	0.4
Free hydroxychloroquine	3.0 ± 0.08	0.2	2.7 ± 0.06	0.1
Free DOX-HCQ (Mix)	2.5 ± 0.04	0.7	2.0 ± 0.05	0.8
Free SLN	3.05 ± 0.07	0.15	2.9 ± 0.04	0.1

## Discussion

Nowadays, the treatment of most diseases caused by intracellular bacteria is difficult, despite widespread advances in the development and the effectiveness of antimicrobial drugs. Most drug combinations in the treatment of brucellosis have shown good results; however, due to the presence of brucellosis inside the host cells, relapse is seen to varying degrees (Edathodu et al., 2021). The aim of this study was to evaluate the therapeutic effect of solid lipid nanoparticles conjugated with cadmium-telluride quantum dot loaded with doxycycline and hydroxychloroquine antibiotics on *Brucella melitensis in vitro* and *in vivo*. The technique used to synthesize nanoparticles in this study was the double emulsion/melt dispersion method. This method is a simple, inexpensive, reproducible technique, requiring organic solvents and limited use of surfactants (Mazur et al., 2019). Due to the fact that the purpose of nanoparticle synthesis in this study was to transfer the drug into macrophages and cells containing bacteria, the properties of nanoparticles were very important. The size of the optimum formulation, PDI, and zeta potential was 214.3 ± 25 nm, 0.385 ± 0.02, and −18.7 mV, respectively. The appropriate size for phagocytosis of nanoparticles by macrophages is between 200 and 600 nm (Kanchan and Panda, 2007). A PDI close to zero indicates uniformity in the size of nanoparticles. High potential zeta increases the stability of nanoparticles during treatment. In the present study, increasing the homogenization time with medium speed, at a temperature of 5–10°C above the melting point of lipid, led to the production of homogeneous micro emulsions. Also, a 5-min increase in homogenization decreased PDI. This finding is consistent with the results of a study by Ghaderkhani et al. (2019) and Liu et al. (2008). In general, the methods of nanoparticle synthesis can be different depending on the purpose of nanoparticle synthesis. Increasing the sonication time can reduce the PDI. In the present study, the nanoparticle size increased after lyophilization. Zeta potential is an important physicochemical parameter that affects the stability of nanostructures. A minimum zeta potential of 10 mV is considered suitable in nanomedicine (Smith et al., 2017). In the present study, zeta potential of nanoparticles was equal to −18.7 mv.

The amount of drug loading and encapsulation in nanoparticles is very important in drug delivery. The findings of the present study showed that the encapsulation and load of doxycycline antibiotics were 95.8 and 19.1%, respectively, while the encapsulation and load of hydroxychloroquine were 92.5 and 16.3%, respectively. Due to the fact that hydroxychloroquine had less solubility than doxycycline, it had a lower load and encapsulation rate. In general, the synthesis method and materials used in the synthesis of nanoparticles have a direct effect on the amount of drug loading and encapsulation. Another important point in the synthesis of nanoparticles is whether the drug is lipophilic or hydrophilic. In the study of Ghaderkhani et al. (2019), rifampin was used to treat brucellosis. Due to the fact that rifampin is a lipophilic drug, a modified microemulsion/sonication method was used to synthesize solid lipid nanoparticle. Their results showed that the loading and encapsulation rate of antibiotics was equal to 97.87 and 34.2%, which is higher than the results of our study. This discrepancy may be due to the structure of the drug because doxycycline is a hydrophilic and rifampin is a lipophilic antibiotic. Various methods have been used to encapsulate hydrophilic drugs, including the modified solvent removal method, hot homogenization and ultrasonication method, and high shear homogenization-ultrasonication method (Patel et al., 2012; Mahardika et al., 2018; Cao et al., 2019). However, the double emulsion method had a higher encapsulation rate than the other methods.

According to the DSC results, it was shown that the drug was molecularly located within the lipid matrix. The disappearance of the peak related to doxycycline and hydroxychloroquine on the DSC thermogram in the synthesized nanoparticle indicates drug interaction with lipid, because lipids tend to dissolve the drug at 65°C. There were no functional peaks or interactions between the nanoparticle components in the FTIR study. The difference between the materials and the solid lipid nanoparticles loaded with the synthesized drug is in the intensity of the peaks. These findings show the stability of the main structure of the drug in the synthesis process. Particle size determination is the best indicator of stability and is used in product evaluation. In solid lipid nanoparticles, the stability is based on the zeta potential (Zielińska et al., 2020). Some of this stability may be due to the preservation of nanoparticles in the original intrinsic state of the particles due to the selection of suitable surfactants. Proper ratio of surfactant and preparation method leads to the synthesis of stable solid lipid nanoparticles. In the present study, the PDI and size was almost constant after 9 months and had little change. But after 9 months, we had a 20.5% increase in nanoparticle size. This increase in size is acceptable because the purpose of using these nanoparticles was phagocytosis by macrophages. Ghaderkhani et al. (2019) investigated the stability of nanoparticles for 9 months after lyophilization, which reported an increase in the size of nanoparticles from 321.7 to 384.3 nm. In the study of Dong et al. (Liu et al., 2014), the stability of the synthesized nanoparticles was investigated for 6 months, in which the zeta potential and drug load were constant but an increase was observed in size.

One of the most important objectives of the present study was the slow release of the drug from the nanodrug. The results showed that it takes 100 h for almost 80% of the drug to be released from the synthesized nanoparticles. The lipid matrix, the concentration of surfactants and co-surfactants, and the parameters involved in the production of solid lipid nanoparticles loaded with doxycycline and hydroxychloroquine are important and effective factors in drug release from nanoparticles. The smaller size of the nanoparticles also reduces the release time of the drug, which is due to the greater contact of the nanoparticle surface.

Well diffusion and MIC findings showed that there was no statistically significant difference in the effect of free drug and synthesized nanodrug on *Brucella melitensis*. Because bacteria are directly exposed to the drug in these methods, free drug had a better effect than the nanodrug in the first 24–48 h. But after 72 h, the zone diameter in well diffusion and minimum inhibitory concentration were almost equal for nanodrug and free drug. In the study of Golmoradzadeh et al. (Fazly Bazzaz et al., 2016), it was shown that solid lipid nanoparticles loaded with rifampin inhibit the biofilm formed by *Staphylococcus epidermidis* at different times and in different concentrations. Another study found that nanodrugs were twice as effective on *Brucella abortus* as free drugs (Ghaderkhani et al., 2019). Unlike well diffusion and MIC tests, in which the bacterium is directly exposed to antibiotics, in the cell study, the bacterium is located inside the macrophage and the antibiotic does not come into direct contact with the bacteria. Therefore, our findings showed that DOX-HCQ-SLN had a better effect on bacteria in macrophages than free drug. Due to the fact that hydroxychloroquine changes the environment inside the phagosome from acidic to alkaline conditions, the slow release of doxycycline and the better efficacy of this antibiotic in alkaline conditions had a significant effect on the elimination of *Brucella melitensis*. In a study by Majzoobi et al. (2018), it was found that the concomitant use of hydroxychloroquine with routine drugs used to treat brucellosis was more effective than the use of routine brucellosis drugs alone. The results of this study showed that the duration of drug release from nanodrug has the greatest effect on bacteria inside the macrophage. Decreased drug release led to increase in the effect of the drug and prevented antibiotic resistance. Nanoparticles were also labeled using cadmium-telluride quantum dot to ensure that nanoparticles entered the macrophages.

Wistar rat was used to evaluate the effect of nanodrug on the animal models. This animal was selected for *in vivo* studies because it is resistant to brucellosis and is easy to work with. *Brucella* proliferation was observed during the first week after infection (Olsen, 2000). It then leads to a systemic and acute infection, in which the infection slowly multiplies so that it enters a chronic phase from the third week and remains in the spleen and liver for the first 5–6 weeks after inoculation (Hosseini et al., 2019b). They can be isolated up to 2 months after infection. The results showed that DOX-HCQ-SLN had a better effect on disease recovery than the free drug in the animal model. After injecting three doses of DOX-HCQ-SLN and Free drug in the acute phase, none of them could completely eliminate the bacteria from the liver and spleen. However, the number of *Brucella melitensis* colonies decreased significantly after treatment with DOX-HCQ-SLN compared to free drug. HCQ-SLN alone did not reduce the number of bacterial colonies in the spleen and liver, but, in general, all of the nanoparticles and even free drug reduced the number of bacterial colonies in the spleen and liver compared to the control group (untreat).

Results were different in the chronic and acute phases. In the DOX-HCQ-SLN–treated rat group, bacteria did not grow on 50% of the spleens and livers from the third dose onward until the last dose (spleens and livers were sterilized). But in the group that received the free drug, none of the spleen and liver was free of bacteria.

Reduction of colony count by drug-loaded lipid nanoparticles in both phases compared to free drug indicates phagocytosis of drug-loaded lipid nanoparticles by macrophages of these two organs. This means that the nanodrug has been able to prevent inactivation of the drug in lysosomal vacuoles within the cell. Due to the nature of the nanodrug matrix (lipid), this compound can cross the walls of the vacuoles and kill bacteria within the cytoplasm. The findings show that the activity and proliferation of bacteria occurs in the acute phase inside and outside the cell, so in this phase, the drug-loaded solid lipid nanoparticles have a similar effect on bacteria as the free drug. On the other hand, in the chronic phase, as the bacterial proliferation decreases, the efficiency of the drug-loaded solid lipid nanoparticles increases at this stage. The main difference between the results of this study and other similar studies is the disease phase. In the present study, the chronic phase of the disease has been investigated, while most studies have examined the treatment in the acute phase of the disease. Lecaroz et al. (2006) examined the microsphere-loaded gentamicin antibiotic on *Brucella melitensis* and showed that the synthesized nanoparticles could reduce the number of bacteria in the acute phase of the disease. Seleem et al. (2009) prepared nanoparticles for loading doxycycline and streptomycin to affect *Brucella melitensis in vivo*, the results of which were similar to those of the present study in the acute phase of the disease. Jain-Gupta et al. (2013) and Imbuluzqueta et al. (2013) conducted a study in the chronic and acute phase of brucellosis, respectively. In line with the present study, the researchers examined gentamicin-containing nanoparticles to reduce the number of *Brucella melitensis* bacteria in the spleen and liver. In another study (Ghaderkhani et al., 2019), oral injection of solid lipid nanoparticles loaded with rifampin resulted in the shelf life of these nanoparticles in the lung, liver, and spleen being up to 10 days and, in plasma, up to 8 days, compared with free rifampin (one to 2 days) in the blood.

## Conclusion

Synthesis of drug-loaded solid lipid nanoparticles using the double emulsion method resulted in optimal size, good encapsulation efficiency, long-term release, and optimal stability. The results of this study show that at similar concentrations and conditions, drug-loaded nanoparticles were more effective than free drug in terms of reducing the number of *Brucella melitensis* bacteria. It was shown that solid lipid nanoparticles are a suitable carrier for the *Brucella melitensis* treatment. Therefore, nanoformulating this drug can increase its effectiveness and eliminate its side effects in free form. Loading antibiotics into solid lipid nanoparticles can increase solubility, reduce cytotoxicity, and improve treatment. However, additional studies are recommended for the preparation of solid lipid nanoparticles containing doxycycline and hydroxychloroquine and other antibiotics involved in the treatment of brucellosis. The overall results of this study suggest that antibiotic-loaded solid lipid nanoparticles pose a promising and alternative strategy for the selective drug delivery of doxycycline to macrophages. The results of this study represent a promising concept that ultimately opens up new avenues in the fight against one of the most pathogenic infectious diseases worldwide. Finally, it is suggested that these nanoparticles be used on animals such as sheep and goats with brucellosis.

## Data Availability

The original contributions presented in the study are included in the article/Supplementary Material, further inquiries can be directed to the corresponding author.
